# Protective vaccinations during pregnancy - adult Poles knowledge in this area

**DOI:** 10.1186/s12889-021-11336-0

**Published:** 2021-07-13

**Authors:** Józefa Dąbek, Oskar Sierka, Halina Kulik, Zbigniew Gąsior

**Affiliations:** 1 Department of Cardiology, Faculty of Health Sciences in Katowice, Medical University of Silesia in Katowice, Ziołowa street 45/47, 40-635 Katowice, Poland; 2 Student Research Group at the Department of Cardiology, Faculty of Health Sciences in Katowice, Medical University of Silesia in Katowice, Ziołowa street 45/47, 40-635 Katowice, Poland; 3 Department of Propaedeutics of Nursing, Faculty of Health Sciences in Katowice, Medical University of Silesia in Katowice, Francuska street 20/24, 40-027 Katowice, Poland

**Keywords:** Preventive vaccinations, Knowledge, Pregnancy

## Abstract

**Background:**

The vaccine is a preparation of biological origin containing antigens that stimulate the body’s immune system to produce acquired immunity. Vaccines can contain killed or “live” (attenuated) microorganisms as well as fragments of these (antigens). Although many vaccines are used routinely in pregnancy to provide a seroprotective immune response for mother, fetus and neonate there is much controversy over their use during this unique time. The aim of the study was to find out about the knowledge of adult Poles on the use of preventive vaccinations during pregnancy.

**Methods:**

The study involved 700 people (100%) aged 18 to 80 years ($\overline{x}$ = 32.16 ± 16.46). Most of the respondents were women (511; 73%). The study consisted of 9 questions about preventive vaccinations of pregnant women and 5 questions about members of the studied group. The aforementioned questions formed the basis of the preparation of the presented article.

**Results:**

A significant part of respondents (322; 46%) did not have knowledge on the topic of safeness of using preventive vaccinations during pregnancy, 196 (28%) respondents believed that such procedure is not safe. Most of the respondents (371; 53%) did not know about the possibility of using “live” vaccines during pregnancy. 14 (2%) of respondents believed that pregnancy should be terminated in case of administration of a “live” vaccine to a pregnant woman. According to 294 (42%) respondents, vaccinations with “live” vaccines should be completed at least 3 months before the planned pregnancy. The subjects were not aware of the issue of post-exposure vaccination against tetanus and rabies among pregnant women. The respondents’ responses were divided on the issue of the safest trimester of pregnancy for vaccine administration. Almost 1/3 of the respondents (203; 29%) indicated the third trimester as the safest for their performance.

**Conclusion:**

The knowledge of the surveyed group, the majority of whom were women, about the use of vaccinations before and during pregnancy was unsatisfactory. There is a need to educate the public about the benefits and risks of performing or avoiding preventive vaccinations during pregnancy.

**Supplementary Information:**

The online version contains supplementary material available at 10.1186/s12889-021-11336-0.

## Background

Vaccines are preparations of biological origin used for active immunoprophylaxis. They contain antigens that stimulate the body’s immune system to produce acquired immunity, known as vaccine immunization. The process of active immunization consists of basic vaccinations (one or more doses given at specified intervals) and booster vaccinations (administration of the next dose of the vaccine to a person who has previously developed acquired immunity) [1].

Pregnancy is a special period in a woman’s life. Developing fetus is genetically different from the organism in which it develops and is considered as a semi-allograft because of the presence of paternal molecules. A pregnant woman’s immune system is seriously affected, by this state and needs to adapt to the presence of mentioned earlier foreign antigens [2–5]. During pregnancy, there is systemic inhibition of responses against the father’s antigens. Cellular immunity, involving T lymphocytes, is weakened and the number of B lymphocytes in peripheral blood decreases, without changing their function. On the other hand, non-specific immunity is strengthened, in which the role is played by monocytes, macrophages and granulocytes, the number of which increases during pregnancy. The amount of regulatory leukocytes T, modulating the course of the immune response, increases already from the first trimester, and the total number of leukocytes in laboratory tests in woman increases gradually during pregnancy, but usually does not exceed 15,000 / mm3 (15 × 10^9^/ l). This condition is called physiological leukocytosis [6, 7]. Even though during pregnancy, changes in non-specific immunity compensate for the decrease in specific cell-type immunity, pregnant women are more susceptible to infections and their complications [8].

Therefore, protective vaccinations against for example COVID-19, influenza or Hepatitis B are intended not only to protect a woman from falling ill but also to protect the developing fetus and ensure its immunity after birth. Their performance became an important global strategy aimed at reduction of mortality and morbidity among pregnant women and developing fetuses, as well as newborns and infants [9–13]. Vaccines should not be withheld among mentioned populations because of pregnancy or breastfeeding but research is always required to determine safety. Various vaccines have been tested for the possibility of their safe use during pregnancy along with the possible effects of their administration [14–20].

Opinions and attitudes for vaccinations vary in society, especially during the COVID-19 pandemic. In Poland, due to the CBOS report from December 2020, only 16% of adult Poles were decided to administer a vaccine against COVID-19. The same report states that in 2020 only 6% of them administered vaccine against influenza [21]. Kilich E. et al. in the conducted meta-analysis identified eight categories of factors that influence maternal vaccination across both qualitative and quantitative studies: accessibility and convenience, personal values and lifestyle, awareness of information regarding the specific vaccine or disease of focus, social influences on vaccine use, emotions related to vaccination, perceptions of vaccine risk, perceptions of vaccine benefit, and personal vaccination history [22]. One of the most important reasons for abandoning vaccination is the fear of women for themselves and their developing children. Research by D’Alessandro A. et al. showed that 23.7% of the women they interviewed believed that vaccination during pregnancy was dangerous for them and the unborn children. On a 10-point scale, women rated this risk from 6.6–7.2. Only 27.9% of the whole sample reported a positive willingness to receive all the recommended vaccinations during pregnancy [23]. A new study, recently released as a preprint on the medRxiv server showed, that acceptability of vaccines against COVID-19 among women was highest for themselves to be vaccinated when not pregnant, with over 8 in 10 of women answering they would likely accept the COVID-19 vaccine. A significantly lower proportion of 6 in 10 women would accept or was leaning towards accepting a COVID-19 vaccine when pregnant, and fewer women answered that they would accept vaccination when pregnant [24]. Health campaigns and programs are other elements that may contribute to the increase in the willingness to take vaccines by women planning or pregnant. Bartolo S. et al. in their studies on the factors influencing the willingness of pregnant women to take influenza vaccine showed that to increase immunization coverage, future health programs should include education about the risk of influenza complications for the developing fetus. It is also extremely important to ensure access to vaccines for any woman who wishes to be vaccinated [25]. Mentioned examples show the importance of the role of the doctors and other members of the medical staff in providing information on immunization. Particular attention should be paid to informing the patients about the safety of vaccination during pregnancy, because, as the research showed, the method and quality of the information provided also affects the willingness of patients to vaccinate [26–30].

There is much controversy around the use of immunization, which distorts the picture of the benefits of vaccination. The aim of the study was to learn about the knowledge of adult Poles on the use of preventive vaccinations during pregnancy. The results of our research may be useful in the preparation of educational programs aimed at improving knowledge in this area and consciously increasing the number of pregnant women vaccinated, and consequently securing the health of pregnant women and their children.

## Methods

Seven hundred people (100%) aged 18 to 80 years ($\overline{x}$ = 35.85 ± 16.2) participated in the study. Most of the respondents were women (500; 71.43%). The study consisted of 9 questions about preventive vaccinations of pregnant women and 5 questions about members of the studied group. The aforementioned questions formed the basis of the preparation of the presented article. Completing the survey was entirely anonymous and voluntary. All respondents gave their informed consent to participate in the study. The questionnaires were collected using the snowball method. The respondents completed the questionnaire by themselves and handed over prepared, clean copies of the questionnaires to their family members and friends. The questionnaires were prepared in the form of traditional paper copies. Each study participant received an identical, unmarked questionnaire with a blank envelope attached. After completing the survey, each respondent placed the completed questionnaire in the aforementioned sealed, unmarked envelope attached to the questionnaire. The sealed envelopes collected from the respondents were placed in a specially prepared, closed box. The above-mentioned box was opened only after filling it in and while entering data into the spreadsheet. Described procedures ensured that the participants and the researchers were unable to identify individual respondents. Questionnaire internal consistency was determined with Cronbach’s alpha coefficient = 0.7597. All methods used in this research were carried out in accordance with relevant guidelines and regulations. In the analysis of the results, all the assessed parameters were presented, both in numerical and percentage values. Results presented in tables were split by age groups, gender, place of current residence level of education and presence of relationship with medical professions. To the respondents declaring the presence of relationship with medical professions authors included responses obtained from doctors, nurses, paramedics and medical students.

## Results

The general characteristics of the studied group are presented in Table 1.

**Table 1:** General characteristics of the studied group (*n* = 700)

Variable			n	%
Gender	Female		500	71,43
	Male		200	28,57
Place of residence	City		612	87,43
	Village		88	12,57
Level of education taking into account gender	Primary	F	16	2,29
		M	18	2,57
	Secondary	F	254	36,29
		M	94	13,43
	Vocational	F	6	0,86
		M	8	1,14
	Higher	F	224	32,00
		M	80	11,43
Presence of reationship with medical professios	Yes		252	36
	No		448	64

**Explanation of abbreviations:** *n* number of respondents, *F* female, *M* male.

More than 70% of the respondents were women. Most of the respondents had average, and the smallest – vocational education. Almost 90% of the respondents lived in the city.

**Fig. 1:**
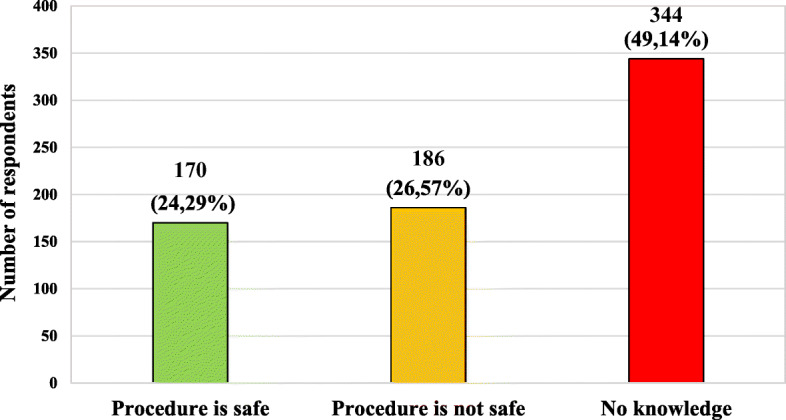
Characteristics of the studied group, taking into account the knowledge of the respondents on the safety of vaccination during pregnancy

The characteristics of the studied group, taking into account the knowledge of the respondents on the safety of vaccination during pregnancy, are presented in Fig. 1. Table 2 presents characteristics of the studied group in the same topic taking into account age, gender, place of current residence, level of education and presence of relationship with medical professions.

**Table 2:** Characteristics of the studied group taking into account respondents’ knowledge on the safety of vaccinations during pregnancy and age, gender, place of current residence, level of education and presence of relationship with medical professions

Possible answers	Procedure is safe		Procedure is **not** safe		No knowledge		
Total (*n* = 700)	170 (24,29%)		186 (26,57%)		344 (49,14%)		
Multiplicity (n;%) of a given group	*n* = 170 (100%)		*n* = 186 (100%)		*n* = 344 (100%)		
**Data**	**n**	**%**	**n**	**%**	**n**	**%**	**Σ**
**Variable**							
**Age (years old)**							
**18–30**	120	70,59 (33,52)	104	55,91 (29,05)	134	38,95 (37,43)	358 (100%)
**31–40**	8	4,71 (12,90)	16	8,60 (25,81)	38	11,05 (61,29)	62 (100%)
**41–50**	32	18,82 (16,49)	44	23,66 (22,68)	118	34,30 (60,82)	194 (100%)
**51–60**	4	2,35 (14,29)	8	4,30 (28,57)	16	4,65 (57,14)	28 (100%)
**> 60**	6	3,53 (10,34)	14	7,53 (24,14)	38	11,05 (65,52)	58 (100%)
**Gender**							
**Female**	112	65,88 (22,40)	138	74,19 (27,60)	250	72,67 (50,00)	500 (100%)
**Male**	58	34,12 (29,00)	48	25,81 (24,00)	94	27,33 (47,00)	200 (100%)
**Place of residence**							
**City**	142	83,53 (23,20)	162	87,10 (26,47)	308	89,53 (50,33)	612 (100%)
**Village**	28	16,47 (31,82)	24	12,90 (27,27)	36	10,47 (40,91)	88 (100%)
**Level of education**							
**Primary**	6	3,53 (17,65)	8	4,30 (23,53)	20	5,81 (58,82)	34 (100%)
**Secondary**	98	57,65 (28,16)	100	53,76 (28,74)	150	43,60 (43,10)	348 (100%)
**Vocational**	2	1,18 (14,29)	4	2,15 (28,57)	8	2,33 (57,14)	14 (100%)
**Higher**	64	37,65 (21,05)	74	39,78 (24,34)	166	48,26 (54,61)	304 (100%)
**Presence of relationship with medical professions**							
**Yes**	108	63,53 (42,86)	68	36,56 (29,98)	76	22,09 (30,16)	252 (100%)
**No**	62	36,47 (13,84)	118	63,44 (26,34)	268	77,91 (59,82)	448 (100%)

**Explanation of abbreviations:** *n* number of respondents.

Almost 50% of the respondents did not have an knowledge on mentioned topic, and about a quarter of respondents believed that the procedure was not safe. In different age groups, the greatest percentage of correct answers was presented by people age 18 to 30. Males marked more answers suggesting the safety of vaccinations during pregnancy than females. Almost 60% of people with no relationship with medical professions declared no knowledge in the discussed matter.

**Fig. 2:**
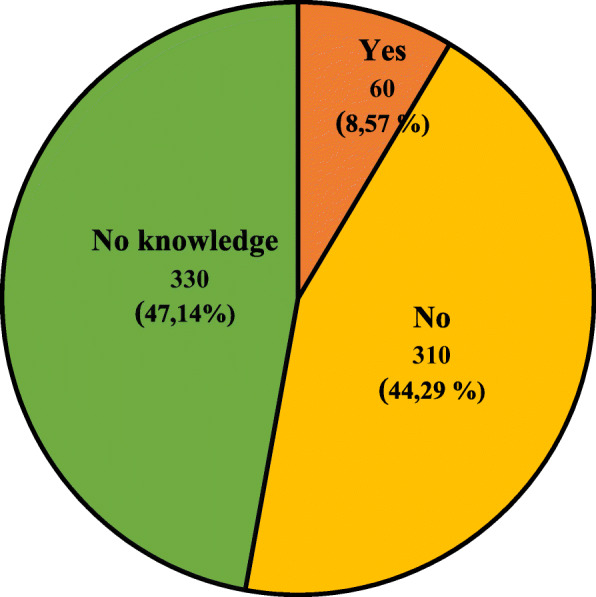
Characteristics of the study group, taking into account the knowledge of the respondents about the possibility of administering a “live” vaccine to a pregnant woman

**Fig. 3:**
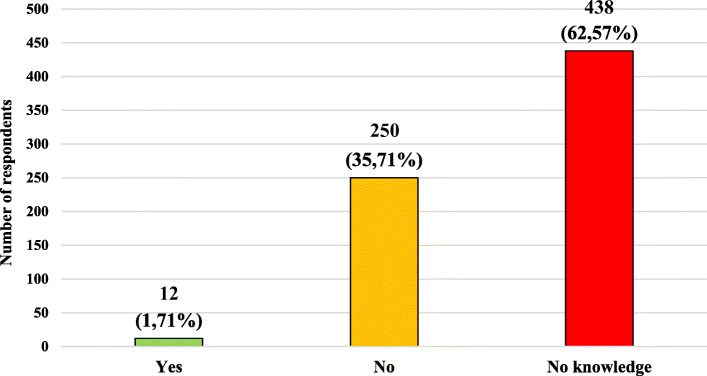
Characteristics of the study group, taking into account the knowledge of the respondents about the necessity or not to terminate pregnancy after administering the vaccine with “live” particles to a pregnant woman

The characteristics of the studied group, taking into account the knowledge of the respondents about the possibility of administering a “live” (attenuated) vaccine to a pregnant woman and necessity or lack of necessity for pregnancy termination after administration of the “live” vaccine, are presented in Figs. 2 and 3. Tables 3 and 4 presents the characteristics of the studied group, taking into account knowledge of the respondents about the possibility of administering “live” (attenuated) vaccine to a pregnant woman and the necessity or lack of necessity for pregnancy termination after administration of the “live” vaccine and age, gender, place of current residence, level of education and relationship with medical professions.

**Table 3:** Characteristics of the studied group, taking into account respondents’ knowledge about the possibility of administering “live” (attenuated) vaccine to a pregnant woman and age, gender, place of current residence, level of education and presence of relationship with medical professions

Possible answers	Yes		No		No knowledge		
Total (*n =* 700)	60 (8,57%)		310 (44,29%)		330 (47,14%)		
Multiplicity (n;%) of a given group	*n* = 60 (100%)		*n* = 310 (100%)		*n* = 330 (100%)		
**Data**	**n**	**%**	**n**	**%**	**n**	**%**	**Σ**
**Variable**							
**Age (years old)**							
**18–30**	48	80,00 (13,41)	184	59,35 (51,40)	126	38,18 (35,20)	358 (100%)
**31–40**	0	0 (0)	32	10,32 (51,61)	30	9,09 (48,39)	62 (100%)
**41–50**	8	13,33 (4,12)	74	23,87 (38,14)	112	33,94 (57,73)	194 (100%)
**51–60**	0	0 (0)	10	3,23 (35,71)	18	5,45 (64,29)	28 (100%)
**> 60**	4	6,67 (6,90)	10	3,23 (17,24)	44	13,33 (75,86)	58 (100%)
**Gender**							
**Female**	36	60,00 (7,20)	230	74,19 (46,00)	234	70,91 (46,80)	500 (100%)
**Male**	24	40,00 (12,00)	80	25,81 (40,00)	96	29,09 (48,00)	200 (100%)
**Place of residence**							
**City**	50	83,33 (8,17)	270	87,10 (44,12)	292	88,48 (47,71)	612 (100%)
**Village**	10	16,67 (11,36)	40	12,90 (45,45)	38	11,52 (43,18)	88 (100%)
**Level of education**							
**Primary**	2	3,33 (5,88)	10	3,23 (29,41)	22	6,67 (64,71)	34 (100%)
**Secondary**	40	66,67 (11,49)	162	52,26 (46,55)	146	44,24 (41,95)	348 (100%)
**Vocational**	2	3,33 (14,29)	4	1,29 (28,57)	8	2,42 (57,14)	14 (100%)
**Higher**	16	26,67 (5,26)	134	43,23 (44,08)	154	46,67 (50,66)	304 (100%)
**Presence of relationship with medical professions**							
**Yes**	40	66,67 (15,87)	140	45,16 (55,56)	72	21,82 (28,57)	252 (100%)
**No**	20	33,33 (4,46)	170	54,84 (37,95)	258	78,18 (57,59)	448 (100%)

**Explanation of abbreviations:** *n* number of respondents.

**Table 4:** Characteristics of the studied group, including respondents’ knowledge about the necessity or lack of necessity for pregnancy termination after administration of the “live” vaccine to a pregnant woman and age, gender, place of current residence, level of education and presence of relationship with medical professions

Possible answers	Yes		No		No knowledge		
Total (*n* = 700)	12 (1,71%)		250 (35,71%)		438 (62,57%)		
Multiplicity (n;%) of a given group	*n* = 12 (100%)		*n* = 250 (100%)		*n* = 438 (100%)		
**Data**	**n**	**%**	**n**	**%**	**n**	**%**	**Σ**
**Variable**							
**Age (years old)**							
**18–30**	10	83,33 (2,79)	168	67,20 (46,93)	180	41,10 (50,28)	358 (100%)
**31–40**	0	0 (0)	22	8,80 (35,48)	40	9,13 (64,52)	62 (100%)
**41–50**	2	16,67 (1,03)	42	16,80 (21,65)	150	34,25 (77,32)	194 (100%)
**51–60**	0	0 (0)	4	1,60 (14,29)	24	5,02 (85,71)	28 (100%)
**> 60**	0	0 (0)	14	5,60 (24,14)	44	10,50 (75,86)	58 (100%)
**Gender**							
**Female**	6	50,00 (1,20)	160	64,00 (32,00)	334	76,26 (66,80)	500 (100%)
**Male**	6	50,00 (3,00)	90	36,00 (45,00)	104	23,74 (52,00)	200 (100%)
**Place of residence**							
**City**	12	100,00 (1,96)	208	83,20 (33,99)	392	89,50 (64,05)	612 (100%)
**Village**	0	0 (0)	42	16,80 (44,73)	46	10,50 (52,27)	88 (100%)
**Level of education**							
**Primary**	2	16,67 (5,88)	12	4,80 (35,29)	20	4,57 (58,82)	34 (100%)
**Secondary**	6	50,00 (1,72)	140	56,00 (40,23)	202	46,12 (58,05)	348 (100%)
**Vocational**	0	0 (0)	6	2,40 (42,86)	8	1,83 (57,14(	14 (100%)
**Higher**	4	33,33 (1,32)	92	36,80 (30,26)	208	47,49 (68,42)	304 (100%)
**Presence of relationship with medical professions**							
**Yes**	4	33,33 (1,59)	148	59,20 (58,73)	100	22,83 (39,68)	252 (100%)
**No**	8	66,67 (1,79)	102	40,80 (22,77)	338	77,17 (75,45)	448 (100%)

**Explanation of abbreviations:** *n* number of respondents.

Over half of the respondents did not know about the possibility of using “live” vaccines during pregnancy. Over 40% of the respondents believed that pregnant women should not be given “live” vaccines, and when they were given, only about 36% said that it was not an indication for termination of pregnancy. Among people with higher education, almost 50% did not know about the possibility of “live” vaccine usage and 70% of the respondents in the mentioned group have no knowledge about the necessity of pregnancy termination after “live” vaccine administration. Only 56% of respondents related to medical professions gave the correct answer to the question about the possibility of “live” vaccine administration. No better results in this group were obtained in the question about necessity of pregnancy termination after “live” vaccine administration. The correct answer was given by 58% of mentioned group respondents.

**Fig. 4:**
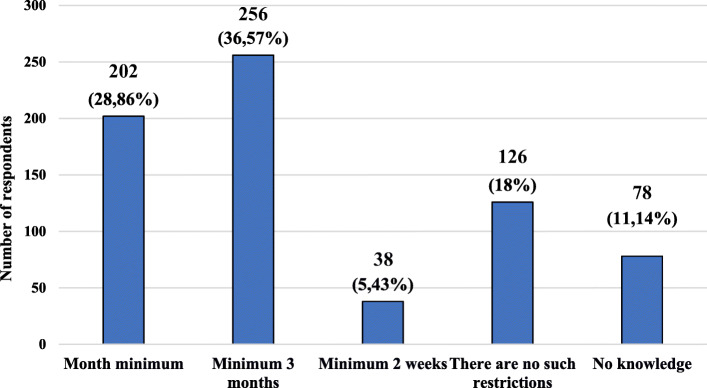
Characteristics of the study group, taking into account the knowledge on the period of time before the planned pregnancy, in which vaccination with “live” vaccines should be completed

The characteristics of the study group, taking into account the respondents’ knowledge about the time before the planned pregnancy should be completed with “live” vaccines, are presented in Fig. 4. Characteristics of the studied group, taking into account the knowledge on the period of time before the planned pregnancy, in which vaccination with “live” vaccines should be completed and age, gender, place of current residence, level of education and presence of relationship with medical professions are presented in Table 5.

**Table 5:** Characteristics of the studied group, taking into account the respondent’s knowledge on the period of time before the planned pregnancy, in which vaccination with “live” vaccines should be completed and age, gender, place of current residence, level of education and presence of relationship with medical professions

Possible answers	Month minimim		Minimum 3 months		Minimum 2 weeks		Thera are no such restrictions		No knowledge		
Total (*n =* 700)	202 (28,86%)		256 (36,57%)		38 (5,43%)		126 (18%)		78 (11,14%)		
Multiplicity (n;%) of a given group	*n* = 202 (100%)		*n* = 256 (100%)		*n* = 38 (100%)		*n* = 126 (100%)		*n* = 78 (100%)		
**Data**	**n**	**%**	**n**	**%**	**n**	**%**	**n**	**%**	**n**	**%**	**Σ**
**Variable**											
**Age (years old)**											
**18–30**	122	60,40 (34,08)	142	55,47 (39,66)	26	38,42 (7,26)	68	53,97 (18,99)	0	0 (0)	358 (100%)
**31–40**	18	8,91 (29,03)	40	15,63 (64,52)	0	0 (0)	4	3,17 (6,45)	0	0 (0)	62 (100%)
**41–50**	36	17,82 (18,56)	48	18,75 (24,74)	8	21,05 (4,12)	24	19,05 (12,37)	78	100,00 (40,21)	194 (100%)
**51–60**	6	2,97 (21,43)	8	3,13 (28,57)	0	0 (0)	14	11,11 (50,00)	0	0 (0)	28 (100%)
**> 60**	20	9,90 (34,48)	18	7,03 (31,03)	4	10,53 (6,90)	16	12,70 (27,59)	0	0 (0)	58 (100%)
**Gender**											
**Female**	138	68,32 (27,60)	196	76,56 (39,20)	28	73,68 (5,60)	80	63,49 (16,00)	58	74,36 (11,60)	500 (100%)
**Male**	64	31,68 (32,00)	60	23,44 (30,00)	10	26,32 (5,00)	46	36,51 (23,00)	20	25,64 (10,00)	200 (100%)
**Place of residence**											
**City**	182	90,10 (29,74)	214	83,59 (34,97)	34	89,47 (5,56)	104	82,54 (16,99)	78	100,00 (12,75)	612 (100%)
**Village**	20	9,90 (22,73)	42	16,71 (47,73)	4	10,53 (4,55)	22	17,46 (25,00)	0	0 (0)	88 (100%)
**Level of education**											
**Primary**	14	6,93 (41,18)	12	4,69 (35,29)	2	5,26 (5,88)	6	4,76 (17,65)	0	0 (0)	34 (100%)
**Secondary**	104	51,49 (29,89)	118	46,09 (33,91)	28	73,68 (8,05)	72	57,14 (20,69)	26	33,33 (7,47)	348 (100%)
**Vocational**	0	0 (0)	6	2,34 (42,86)	0	0 (0)	8	6,35 (57,14)	0	0 (0)	14 (100%)
**Higher**	84	41,58 (27,63)	120	46,88 (39,47)	8	21,05 (2,63)	40	31,75 (13,16)	52	66,67 (17,11)	304 (100%)
**Presence of relationship with medical professions**											
**Yes**	80	39,66 (31,75)	92	35,94 (36,51)	24	63,16 (9,52)	54	42,86 (21,43)	2	2,56 (0,79)	252 (100%)
**No**	122	60,40 (27,23)	164	64,06 (36,61)	14	36,84 (3,13)	72	57,14 (16,07)	76	97,44 (16,96)	448 (100%)

**Explanation of abbreviations:** *n* number of respondents.

Most respondents believed that vaccinations with “live” vaccines should be completed at least 3 months before the planned pregnancy, while almost 1/5 of respondents believed that there are no time limits in this regard. In the study group divided by age, only people in the age group 41–50 showed a lack of knowledge. The greatest amount of good answers among people categorized by level of education was given by people with primary education. Only 28% of females chose answers “Minimum month”, moreover only about 30% of respondents related with medical professions gave the correct answer to this question.

**Fig. 5:**
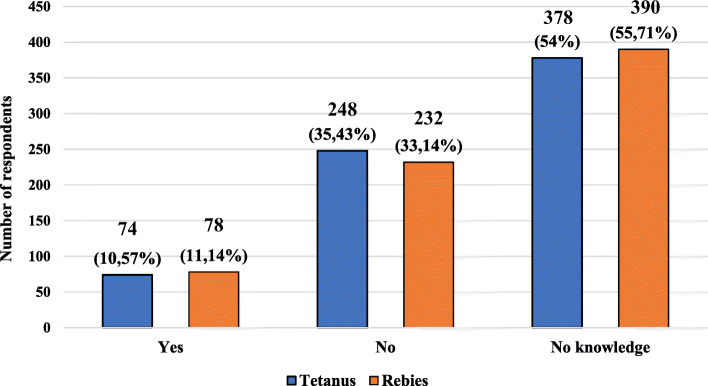
Characteristics of the studied group, taking into account the respondents knowledge on the possibility of post-exposure vaccinations in the event of exposure of a pregnant woman to tetanus and / or rabies

The characteristics of the studied group, taking into account the knowledge of the respondents’ about the possibility of vaccination in pregnant women after exposure to tetanus and rabies, are presented in Fig. 5. The characteristics of the studied group, taking into account the knowledge of the respondents about the possibility of vaccination in pregnant women after exposure to tetanus and rabies and age, gender, place of current residence, level of education and presence of relationship with medical professions are presented in Tables 6 and 7.

**Table 6:** Characteristics of the studied group, including the respondents’ knowledge on the possibility of post-exposure vaccinations in the event of exposure of a pregnant woman to tetanus and age, gender, place of current residence, level of education and presence of relationship with medical professions

Possible answers	Yes		No		No knowledge		
Total (*n =* 700)	74 (10,57%)		248 (35,43%)		378 (54,00%)		
Multiplicity (n;%) of a given group	*n* = 74 (100%)		*n* = 248 (100%)		*n* = 378 (100%)		
**Data**	**n**	**%**	**n**	**%**	**n**	**%**	**Σ**
**Variable**							
**Age (years old)**							
**18–30**	48	64,86 (13,41)	164	66,13 (54,81)	146	38,62 (40,78)	358 (100%)
**31–40**	0	0 (0)	18	7,26 (29,03)	44	11,64 (70,97)	62 (100%)
**41–50**	18	24,32 (9,28)	50	20,16 (25,77)	126	33,33 (64,95)	194 (100%)
**51–60**	4	5,41 (14,29)	6	2,42 (21,43)	18	4,76 (64,29)	28 (100%)
**> 60**	4	5,41 (6,90)	10	4,03 (17,24)	44	11,64 (75,86)	58 (100%)
**Gender**							
**Female**	46	62,16 (9,20)	174	70,16 (34,80)	280	74,07 (56,00)	500 (100%)
**Male**	28	37,84 (14,00)	74	29,84 (37,00)	98	25,93 (49,00)	200 (100%)
**Place of residence**							
**City**	58	78,38 (2,81)	214	86,29 (37,54)	340	89,95 (59,65)	612 (100%)
**Village**	16	21,62 (18,18)	34	13,71 (38,64)	38	10,05 (43,18)	88 (100%)
**Level of education**							
**Primary**	10	13,51 (29,41)	4	1,61 (11,76)	20	5,29 (58,82)	34 (100%)
**Secondary**	34	45,95 (9,77)	142	57,26 (4,80)	172	45,50 (49,43)	348 (100%)
**Vocational**	0	0 (0)	4	1,61 (28,57)	10	2,65 (71,43)	14 (100%)
**Higher**	30	40,45 (3,87)	98	39,52 (32,24)	176	46,56 (57,89)	304 (100%)
**Presence of relationship with medical professions**							
**Yes**	26	35,14 (10,32)	158	63,71 (62,70)	68	17,99 (26,98)	252 (100%)
**No**	48	64,86 (10,71)	90	36,29 (20,09)	310	82,01 (69,20)	448 (100%)

**Explanation of abbreviations:** *n* number of respondents.

**Table 7:** Characteristics of the studied group, including the respondents’ knowledge on the possibility of post-exposure vaccinations in the event of exposure of a pregnant woman to rabies and age, gender, place of current residence, level of education and presence of relationship with medical professions

Possible answers	Yes		No		No knowledge		
Total (*n =* 700)	78 (11,14%)		232 (33,14%)		390 (55,71%)		
Multiplicity (n;%) of a given group	*n =* 78 (100%)		*n* = 232 (100%)		*n* = 390 (100%)		
**Data**	**n**	**%**	**n**	**%**	**n**	**%**	**Σ**
**Variable**							
**Age (years old)**							
**18–30**	52	66,67 (14,53)	152	65,52 (42,46)	154	39,49 (43,02)	358 (100%)
**31–40**	0	0 (0)	16	6,90 (25,81)	46	11,79 (74,19)	62 (100%)
**41–50**	18	23,08 (9,28)	52	22,41 (26,80)	124	31,79 (63,92)	194 (100%)
**51–60**	2	2,56 (7,14)	4	1,72 (14,29)	22	5,64 (78,57)	28 (100%)
**> 60**	6	7,69 (10,34)	8	3,45 (13,79)	44	11,28 (75,86)	58 (100%)
**Gender**							
**Female**	50	64,10 (10,00)	158	68,10 (31,60)	292	74,87 (58,40)	500 (100%)
**Male**	28	35,90 (14,00)	74	31,90 (37,00)	98	25,13 (49,00)	200 (100%)
**Place of residence**							
**City**	66	84,62 (10,78)	194	83,62 (31,70)	352	90,26 (57,52)	612 (100%)
**Village**	12	15,38 (13,64)	38	16,38 (43,18)	38	9,74 (43,18)	88 (100%)
**Level of education**							
**Primary**	10	12,82 (29,41)	8	3,45 (32,53)	16	4,10 (47,06)	34 (100%)
**Secondary**	42	53,88 (12,07)	128	55,17 (36,78)	178	45,64 (51,15)	348 (100%)
**Vocational**	2	2,56 (14,29)	4	1,72 (28,57)	8	2,05 (57,14)	14 (100%)
**Higher**	24	30,77 (7,89)	92	39,66 (30,26)	188	48,21 (61,84)	304 (100%)
**Presence of relationship with medical professions**							
**Yes**	32	41,03 (12,70)	140	60,34 (55,56)	80	20,51 (31,75)	252 (100%)
**No**	46	58,97 (10,27)	92	39,66 (20,54)	310	79,49 (69,20)	448 (100%)

**Explanation of abbreviations:** *n* number of respondents.

More than half of the respondents did not know about post-exposure vaccination against tetanus and rabies. In the case of the above-mentioned vaccinations, more than 30% of respondents believed that pregnancy was a contraindication to their vaccination. In the studied group, no knowledge was declared by 56% of women in case of post-exposure vaccination against tetanus and 58% in case of post-exposure vaccination against rabies. Among men, 49% of them declared no knowledge in both areas. Almost 58% of respondents with higher education have no knowledge about postexposure tetanus vaccination and almost 62% about postexposure rabies vaccination. In a group of respondents with no relationship with medical professions, almost 70% did not know about post-exposure vaccination against tetanus and rabies.

**Fig. 6:**
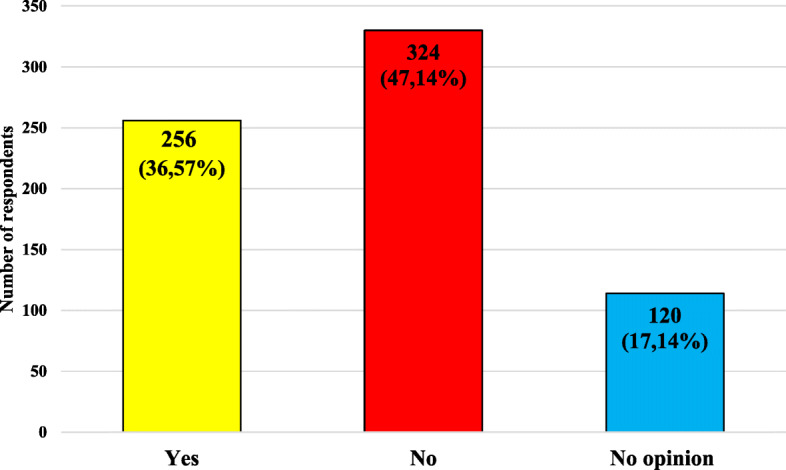
Characteristics of the studied group, taking into account the respondents’ knowledge on the subject of advising pregnant women or women planning to become pregnant to be vaccinated against influenza

The characteristics of the studied group, including the respondents’ knowledge on recommending vaccination against influenza to pregnant women or planning a pregnancy are presented in Fig. 6. Characteristics of the studied group, taking into account the respondents knowledge on the subject of advising pregnant women or women planning to become pregnant to be vaccinated against influenza and age, gender, place of current residence, level of education and presence of relationship with medical professions are presented in Table 8.

**Table 8:** Characteristics of the studied group, taking into account the respondents’ knowledge on the subject of advising pregnant women or women planning to become pregnant to be vaccinated against influenza and age, gender, place of current residence, level of education and presence of relationship with medical professions

Possible answers	Yes		No		No knowledge		
Total (*n =* 700)	256 (36,57%)		324 (47,14%)		120 (17,14%)		
Multiplicity (n;%) of a given group	*n =* 256 (100%)		*n* = 324 (100%)		*n* = 120(100%)		
**Data**	**n**	**%**	**n**	**%**	**n**	**%**	**Σ**
**Variable**							
**Age (years old)**							
**18–30**	172	67,19 (48,04)	160	49,38 (44,69)	26	21,67 (7,26)	358 (100%)
**31–40**	12	4,69 (19,35)	48	14,81 (77,42)	2	1,67 (3,23)	62 (100%)
**41–50**	46	17,97 (23,71)	76	23,46 (39,18)	72	60,00 (37,11)	194 (100%)
**51–60**	10	3,91 (35,71)	4	1,23 (14,29)	14	11,67 (50,00)	28 (100%)
**> 60**	16	6,25 (27,59)	36	11,11 (62,07)	6	5,00 (10,34)	58 (100%)
**Gender**							
**Female**	168	65,63 (33,60)	244	75,31 (48,80)	88	73,33 (17,60)	500 (100%)
**Male**	88	34,38 (44,00)	80	24,69 (40,00)	32	26,67 (16,00)	200 (100%)
**Place of residence**							
**City**	214	83,59 (34,97)	286	88,27 (46,73)	112	93,33 (18,30)	612 (100%)
**Village**	42	16,41 (47,73)	38	11,73 (43,18)	8	6,67 (9,09)	88 (100%)
**Level of education**							
**Primary**	14	5,47 (41,18)	10	3,09 (29,41)	10	8,33 (29,41)	34 (100%)
**Secondary**	154	60,16 (44,25)	146	45,06 (41,95)	48	40,00 (13,79)	348 (100%)
**Vocational**	4	1,56 (28,57)	8	2,47 (57,14)	2	1,67 (14,29)	14 (100%)
**Higher**	84	32,81 (27,63)	160	49,38 (52,63)	60	50,00 (19,74)	304 (100%)
**Presence of relationship with medical professions**							
**Yes**	136	53,13 (53,97)	100	30,89 (39,68)	16	13,33 (6,35)	252 (100%)
**No**	120	46,88 (26,79)	224	69,14 (50,00)	104	86,67 (23,21)	448 (100%)

**Explanation of abbreviations:** *n* number of respondents.

Almost 50% of respondents’ denied the need to recommend influenza vaccination to pregnant women and women planning to become pregnant. The highest rate of correct answers was presented by the group of people 18–30 years. Only 54% of respondents with the presence of relationship with medical professions and 28% of people with higher education would recommend vaccination against influenza to a pregnant woman.

**Fig. 7:**
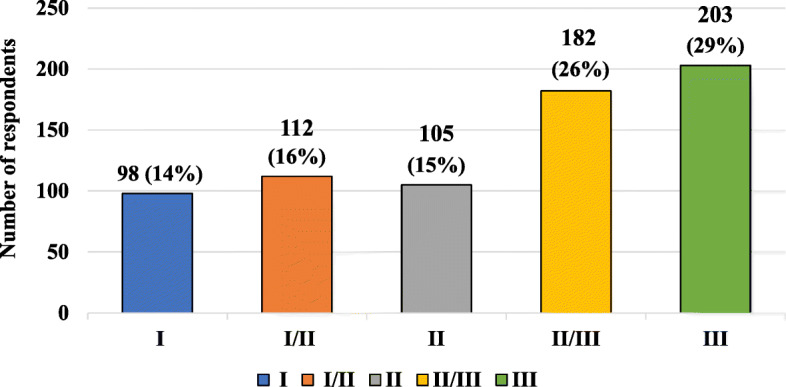
Characteristics of the studied group, taking into account the knowledge of the respondents’ about the safest trimester of pregnancy to carry out preventive vaccinations

The characteristics of the studied group, including the respondents’ knowledge about the safest trimester of pregnancy for vaccination in pregnant women, are presented in Fig. 7. Table 9 presents characteristics of the studied group, taking into account the knowledge of the respondents about the safest trimester of pregnancy to carry out preventive vaccinations and age, gender, place of current residence, level of education and presence of relationship with medical professions.

**Table 9:** Characteristics of the studied group taking into account the knowledge of the respondents about the safest trimester of pregnancy to carry out preventive vaccinations and age, gender, place of current residence, level of education and presence of relationship with medical professions

Possible answers	I		I/II		II		II/III		III		
Total (*n =* 700)	98 (14%)		112 (16%)		105 (15%)		182 (26%)		203 (29%)		
Multiplicity (n;%) of a given group	*n* = 98 (100%)		*n* = 112 (100%)		*n* = 105 (100%)		*n* = 182 (100%)		*n* = 203 (100%)		
**Data**	n	%	n	%	n	%	n	%	n	%	**Σ**
**Variable**											
**Age (years old)**											
**18–30**	50	51,02 (13,97)	78	69,64 (21,79)	56	53,33 (15,64)	104	57,14 (29,05)	70	34,48 (19,55)	358 (100%)
**31–40**	9	9,18 (14,52)	6	5,36 (9,68)	10	9,52 (16,13)	20	10,99 (32,26)	17	8,37 (27,42)	62 (100%)
**41–50**	27	27,55 (13,92)	18	16,07 (9,28)	17	16,19 (8,76)	40	21,98 (20,62)	92	45,32 (47,42)	194 (100%)
**51–60**	2	2,04 (7,14)	4	3,57 (14,29)	6	5,71 (21,43)	6	3,30 (21,43)	10	4,93 (35,71)	28 (100%)
**> 60**	10	10,20 (17,24)	6	5,36 (10,43)	16	15,24 (27,59)	12	6,59 (20,69)	14	6,90 (24,14)	58 (100%)
**Gender**											
**Female**	65	66,33 (13,00)	87	77,68 (17,40)	84	80,00 (16,80)	138	75,82 (27,60)	126	62,07 (25,20)	500 (100%)
**Male**	33	33,67 (16,50)	25	22,32 (12,50)	21	20,00 (10,50)	44	24,18 (22,00)	77	37,93 (38,50)	200 (100%)
**Place of residence**											
**City**	86	87,76 (14,05)	98	87,50 (16,01)	89	84,76 (14,54)	154	84,62 (25,16)	185	91,13 (30,23)	612 (100%)
**Village**	12	12,24 (13,64)	14	12,50 (15,91)	16	15,24 (18,18)	28	15,38 (31,82)	18	8,87 (20,45)	88 (100%)
**Level of education**											
**Primary**	6	6,12 (17,65)	9	8,04 (26,47)	4	3,81 (11,76)	13	7,14 (38,24)	2	0,99 (5,88)	34 (100%)
**Secondary**	45	45,92 (12,93)	64	57,14 (18,39)	46	43,81 (13,22)	98	53,85 (28,16)	95	46,80 (27,30)	348 (100%)
**Vocational**	0	0 (0)	0	0 (0)	2	1,90 (14,29)	2	1,11 (14,29)	10	4,93 (71,43)	14 (100%)
**Higher**	47	47,96 (15,46)	39	34,80 (12,83)	53	50,48 (17,43)	69	37,91 (22,70)	96	47,29 (31,58)	304 (100%)
**Presence of relationship with medical professions**											
**Yes**	24	24,49 (9,52)	48	42,86 (19,05)	36	34,29 (14,29)	81	44,51 (32,14)	63	31,03 (25,00)	252 (100%)
**No**	74	75,51 (16,52)	64	57,14 (14,29)	69	65,71 (15,40)	101	55,49 (22,54)	140	68,97 (31,25)	448 (100%)

**Explanation of abbreviations:** *n* number of respondents.

Respondents’ answers regarding the trimester of pregnancy in which it is safest to vaccinate were divided. Most respondents indicated the third trimester as the safest time for their performance. In the study group divided by age, people in the group 41–50 years have the highest rate of answers suggesting the third trimester as the safest. More than 30% of people living in the cities and people with higher education chose III trimester as the safest to perform vaccinations. Among men, only 39% of them declared that the III trimester is the safest to perform vaccinations. In a group of respondents with the presence of relationship with medical professions, only 25% chose III trimester as the safest for vaccination performing.

**Fig. 8:**
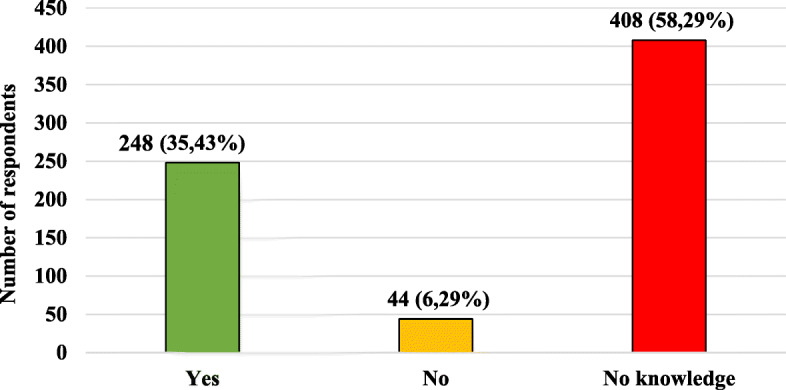
Characteristics of the studied group taking into account knowledge on the possibility of using passive immunization (specific immunoglobulin) in women and non-specific in the case of exposure to infectious diseases such as measles, chickenpox or rubella

Figure 8 shows characteristics of the study group, taking into account respondents’ knowledge about the possibility of administering specific and non-specific immunoglobulins to pregnant women in the case of exposure to infectious diseases such as measles, chickenpox and rubella, to reduce the possibility of suffering from them. Table 10 presents characteristics of the studied group, taking into account the knowledge of the respondents about the possibility of using passive immunization (specific immunoglobulin) in women and non-specific in the case of exposure to infectious diseases such as measles, chickenpox or rubella and age, gender, place of current residence, level of education and presence of relationship with medical professions.

**Table 10:** Characteristics of the studied group, taking into account the knowledge of the respondents about the possibility of using passive immunization (specific immunoglobulin) in women and non-specific in the case of exposure to infectious diseases such as measles, chickenpox or rubella and age, gender, place of current residence, level of education and presence of relationship with medical professions

Possible answers	Yes		No		No knowledge		
Total (*n =* 700)	248 (35,43%)		44 (6,29%)		408 (58,29%)		
Multiplicity (n;%) of a given group	*n =* 248 (100%)		*n* = 44 (100%)		*n* = 408 (100%)		
**Data**	**n**	**%**	**n**	**%**	**n**	**%**	**Σ**
**Variable**							
**Age (years old)**							
**18–30**	180	72,58 (50,28)	22	50,00 (6,15)	156	38,24 (43,58)	358 (100%)
**31–40**	14	5,65 (22,58)	2	4,55 (3,23)	46	11,27 (74,19)	62 (100%)
**41–50**	46	18,55 (23,71)	12	27,27 (6,19)	136	33,33 (70,10)	194 (100%)
**51–60**	4	1,61 (14,29)	0	0 (0)	24	5,88 (85,71)	28 (100%)
**> 60**	4	1,64 (4,90)	8	18,18 (13,79)	46	11,27 (79,31)	58 (100%)
**Gender**							
**Female**	184	74,19 (36,80)	32	72,73 (6,40)	284	69,61 (56,80)	500 (100%)
**Male**	64	25,81 (32,00)	12	27,27 (6,00)	124	30,39 (62,00)	200 (100%)
**Place of residence**							
**City**	216	87,10 (35,29)	38	86,39 (6,21)	358	87,75 (58,50)	612 (100%)
**Village**	32	12,90 (37,65)	3	6,82 (3,53)	50	12,25 (58,82)	88 (100%)
**Level of education**							
**Primary**	14	5,65 (41,18)	2	4,55 (5,88)	18	4,41 (52,94)	34 (100%)
**Secondary**	144	58,06 (41,38)	24	54,55 (6,90)	180	44,12 (51,72)	348 (100%)
**Vocational**	2	0,81 (14,29)	2	4,55 (14,29)	10	2,45 (71,43)	14 (100%)
**Higher**	88	35,48 (28,95)	16	36,36 (5,26)	200	49,02 (65,79)	304 (100%)
**Presence of relationship with medical professions**							
**Yes**	150	60,48 (59,52)	18	40,91 (7,14)	84	20,59 (33,33)	252 (100%)
**No**	98	39,52 (21,88)	26	59,09 (5,80)	324	79,41 (72,32)	448 (100%)

**Explanation of abbreviations:** *n* number of respondents.

Almost 60% of the respondents did not know about specific and non-specific immunoglobulins used in pregnant women. 1/3 of respondents related to medical professions did not know in this area. More correct answers were given by women compared to men and people with the presence of relationship with medical professions than people with no relationship with medicine.

## Discussion

The presented results reveal how challenging is the topic of preventive vaccinations during pregnancy for adults. In line with the recommendations of the Advisory Committee on Immunization Practices (ACIP) at the Centers for Disease Control and Prevention and the American College of Obstetrics and Gynecology (ACOG) as well as the guidelines in force in Poland, pregnant women should be vaccinated if a safe vaccine is available and there is a risk of exposure of the woman to a disease that threatens herself and/or the child [31]. The authors of various publications unanimously suggest that immunization not only protects the mother but also plays a key role in the protection of the fetus and infants until they develop a fully functional immune system [32–36]. Antibodies produced after vaccination are transmitted through the placenta from around the 13th week of pregnancy [11]. Their deficiency makes infants susceptible to severe diseases [34]. Research conducted by Healy M. et al. showed that 83,9% of pregnant women examined by them had knowledge about the safeness of recommended vaccinations during pregnancy [35]. The study carried out by Bartolo S. et al. on 2069 women, showed that 827 (40%) questioned women did not know that influenza can lead to severe adverse outcomes for the mother, and 960 (46%) did not know about possible severe adverse outcomes for the baby [36]. Out of the 700-person group of respondents, as many as 75% did not know about the possibility of immunization in pregnant women or gave the wrong answer. Worryingly, half of the women were unaware of the safeness of immunization performance during pregnancy. Equally disturbing is the fact that the lack of knowledge in the discussed topic was reported by over 30% of people declaring a presence of relationship with medical professions. A much greater percentage of people declaring a lack of knowledge were people with secondary (43.10%) and higher (54.61%) education.

Despite the possibility of carrying out preventive vaccinations in pregnancy, one should remember indications and contraindications for their administration. In the ACOG guidelines and according to S. Chang et al. during pregnancy, vaccination with “live” vaccines should be avoided [31, 37]. A meta-analysis by A. Laris-González et al. shows that only the smallpox vaccine was associated with the risk of developing pregnancy complications, while S. Chang et al. concluded that all “live” vaccines pose a risk to the fetus [37, 38]. In the own study, more than half of the respondents answered incorrectly (8.57%) or did not know (47.14%) about the possibility of vaccinating with a “live” vaccine during pregnancy. Despite so many wrong answers, 44% of respondents answered this question correctly. Among people with no connection to medicine, it was almost 40% of the respondents. It is possible that the answers obtained did not result directly from their knowledge, but rather from the assumptions made by the respondents. A “live” vaccine might have been associated with a fully functional microorganism that can cause disease to the mother, fetus and newborn, and affect their future life. This is probably the reason for a large number of respondents indicating the answer denying giving the pregnant woman a “live” vaccine.

It is also possible to vaccinate a pregnant woman (often unconsciously) with a “live” vaccine or to become pregnant before the expiry of the recommended by both ACIP and the Polish 2020 Immunization Calendar, 4 weeks after vaccination with a “live” vaccine [31, 39]. ACIP in its guidelines clearly stated that this situation was not an indication for termination of pregnancy [31]. However, among the respondents, there was a group claiming that administration of the “live” vaccine to a pregnant woman is an indication for termination of pregnancy (1.71%), and over 60% of the respondents had no knowledge in this regard. Only 202 (28.86%) respondents gave the correct answer (4 weeks) to the question about the time that should elapse between the administration of a “live” vaccine and pregnancy. Better knowledge was presented by people with the presence of a relationship with medical professions in both cases. Yet, almost 2% of the mentioned group claimed that after “live” vaccine termination, the pregnancy should be terminated and almost 40% have no knowledge of this subject.

According to the ACIP recommendations [31], vaccination against tetanus should be performed between the 27th and 34th week of pregnancy, regardless of whether the woman was previously vaccinated or not. If a pregnant woman is exposed to *Clostridium tetani*, she may be given Tdap vaccine with reduced pertussis and diphtheria antigens, even if the exposure took place at a different time than the preferred time for administration of the vaccine. The results of the research by M. McMillan et al. [40] showed that the combined vaccine against diphtheria, tetanus and whooping cough used in the second and third trimesters caused no clinically significant harm to either the fetus or the newborn. Pregnancy should also not be considered a contraindication to post-exposure administration of a vaccine against rabies to pregnant women [41]. In line with the observations, also N. Faucette et al. [42] an inactivated rabies virus vaccine may be administered to pregnant women if exposed. Research by D’Alessandro A et al. showed that only 7% of the women surveyed by them knew about the need to vaccinate or administer a tetanus stimulant before a planned pregnancy [23]. Over half of the respondents in own research did not know about post-exposure vaccinations against both tetanus (54%) and rabies (55.71%). A slightly higher result than that obtained by D’Alessandro et al. was obtained among women from own research (9.20%). It is disturbing that over 61% of respondents with higher education and 51% with secondary education declared a lack of knowledge about the possibility of administering the vaccine against rabies in the event of exposure to rabies. For both rabies and tetanus vaccines, just over 10% of those associated with the medicine answered correctly.

Influenza is one of the most frequently observed viral diseases in the world. It occurs seasonally. It is transmitted by airborne droplets and can cause serious complications. In the authors’ research conducted in 2011 in a group of adult patients of a GP clinic, out of 312 patients, only 134 (42.49%) were vaccinated against seasonal influenza. In this group, 78 (58.20%) patients were vaccinated regularly [43]. Researchers C. Nunes et al. showed that the influenza virus may contribute to the increased susceptibility to infection with bacteria such as *Streptococcus pneumoniae, Haemophilus influenzae and Staphylococcus aureus* [44]. Pregnant women are included by WHO in the group of high risk of developing influenza and particularly predisposed to carry out the vaccination. Infection with influenza virus may cause hospitalization, serious circulatory and respiratory disorders, as well as miscarriages and infections of newborns [45]. According to Carlson A., complications most often develop in the second or third trimester of pregnancy [46]. Both the ACIP and WHO guidelines do not specify a precise period in which a woman should get the flu vaccine. Therefore, she can be vaccinated both before and during pregnancy, regardless of whether the woman gets into it before, during or after the “flu season” [47]. The best time for vaccination, according to Mak D. et al. is the third trimester when the best maternal-to-fetal transfer of antibodies is observed. The problem, in this case, is the time of the first and second trimesters, when the woman is not protected against influenza [48]. Research by Vishram B. et al. found that 73% of surveyed health care members would recommend influenza vaccination to pregnant women [49]. In contrast, out of 261 women tested by Blanchard-Rohner G. et al., 119 (46%) knew that influenza vaccination is recommended for pregnant women [50]. In own research, a small group of respondents (36.57%) correctly answered the above-mentioned question. Moreover, only about 54% of current and future members of the health care system would recommend vaccination against influenza during pregnancy and the percentage of women who knew that influenza vaccination is recommended for pregnant women was significantly lower (33%) among respondents from own study compared to respondents from the research of Blanchard-Rohner G et.al. Once again, we found out about the lack of public awareness of vaccination during pregnancy. Presented results may indicate an insufficient message of social campaigns concerning regular vaccination against influenza and other vaccine-controlled diseases. Moreover, the results showed that people who should constantly strive to raise public awareness of vaccination did not have complete knowledge in this regard.

ACIP and WHO recommendations [31, 45] state that if vaccinations are not necessary, their administration during pregnancy should be avoided. However, if there is a need to vaccinate a pregnant woman, the more advanced gestation stage, the safer the vaccination is. In the asked question great deal of the respondents inclined to the extreme answers, i.e. the turn of the second and third trimester and the third trimester of pregnancy. These answers may suggest possessing some knowledge in this regard, but the percentage of responses pointing to other periods of pregnancy is still high. This situation could have been caused by a lack of interest in this topic in all the presented groups. The problem is even more important due to the fact that the majority of the studied group were women, who should have more knowledge in this subject.

The guidelines of the Australian Association of Infectious Diseases [51] and the American CDC [52] indicate that if there is exposure to diseases such as measles, chickenpox, rubella, pregnancy is not a contraindication to the post-exposure administration of specific and/or non-specific immunoglobulins to the woman. The question of administering specific and non-specific immunoglobulins to pregnant women during the exposure to measles, varicella and rubella was the most difficult for the study group. Giving the correct answer required specialist knowledge, hence more than 60% of the respondents did not give the correct answer. Among the people who gave the largest number of correct answers were people with the presence of relationship to medical professions compared to people with no such relationship and people aged 18–30 in relation to other age groups.

Further research of the public knowledge on immunization, not only during pregnancy, should focus on the reasons for the lack of knowledge on the topic in question. Particular attention should also be paid to knowledge gaps among people associated with medicine. Actions should be implemented to improve the knowledge of these group members. It is also worth considering strengthening activities to promote awareness about vaccinations in society.

In connection with the emerging anti-vaccine movements around the world and access to information that is often not based on scientific evidence, the role of doctors, both primary care and specialists, is of great importance in making the public aware of the benefits of immunization, both during pregnancy and beyond.

## Conclusions

The knowledge of the surveyed group, the majority of whom were women, about the use of vaccinations before and during pregnancy was insufficient. There is a need for continuous education of the society about the benefits and risks of performing or avoiding preventive vaccinations during pregnancy.

## Supplementary Information

**Additional file 1.**

## Data Availability

The datasets used and analysed during the current study are available from the corresponding author on reasonable request.

## Abbreviations

F: Female
M: Male
n: Numer of respondents
