# Supplementary material for: Protective vaccinations during pregnancy - adult Poles knowledge in this area
Source: BMC Public Health. 2021 Jul 13;21:1392. doi: 10.1186/s12889-021-11336-0 (PMC8276846; doi:10.1186/s12889-021-11336-0)
Supplement: Supplementary file 1 — Additional file 1. [file 12889_2021_11336_MOESM1_ESM.docx]

**Suppl.1.** Questions addressed to adult Poles about their knowledge and opinions on the topic of vaccination during pregnancy asked via questionaire with possible selectable answers.

| Asked questions: |
| --- |
| 1. Please, enter your age: ……………………………………. |
| 1. Choose gender 2. Female 3. Male |
| 1. Where do you live?   a) City  b) Village |
| 1. Please, mark the level of your education; 2. Primary 3. Secondary 4. Vocational 5. Higher |
| 1. Are you a doctor, nurse, paramedic or medical student? 2. Yes 3. No |
| 1. Is it safe to have immunization during pregnancy? 2. Procedurę is safe. 3. Procedure is not safe. 4. I do not know. |
| 1. Can a pregnant woman be given a "live" (atenuated) vaccine? 2. Yes 3. No 4. I do not know. |
| 1. Is the administration of a "live" vaccine to a pregnant woman an indication for termination of pregnancy? 2. Yes 3. No 4. I do not know. |
| 1. What time should pass between getting pregnant and getting the "live" vaccine? 2. Month minimum 3. Minimum 3 months 4. Minimum 2 weeks 5. There are no such restrictions 6. I do not know |
| 1. Can a pregnant woman be vaccinated against tetanus in case of exposure? 2. Yes 3. No 4. I do not know. |
| 1. Can a pregnant woman be vaccinated against rabies in case of exposure? 2. Yes 3. No 4. I do not know. |
| 1. Should a woman who is pregnant or planning to become pregnant be advised to vaccinate against flu? 2. Yes 3. No 4. I do not know. |
| 1. Which trimester is safest for immunization? 2. I 3. I/II 4. II 5. II/III 6. III |
| 1. In the case of infectious diseases such as measles, chicken pox, rubella in pregnant women, in order to reduce the possibility of become ill, is it possible to use passive immunization? (specific and non-specific immunoglobulins). 2. Yes 3. No 4. I do not know. |
